# New Measures of Economic Insecurity Reveal its Expansion Into EU Middle Classes and Welfare States

**DOI:** 10.1007/s11205-021-02709-4

**Published:** 2021-05-20

**Authors:** Costanzo Ranci, Jason Beckfield, Laura Bernardi, Andrea Parma

**Affiliations:** 1 Department of Architecture and Urban Studies, Politecnico Di Milano, via Bonardi, 3 20133 Milan, Italy; 2 Department of Sociology, Harvard University, 550 William James Hall | 33 Kirkland Street, Cambridge, MA 02138 USA; 3 Institut des sciences sociales and LIVES Center, Université de Lausanne, Quartier UNIL-Mouline, Bâtiment Géopolis, CH-1015 Lausanne, Switzerland; 4 Department of Architecture and Urban Studies, Politecnico Di Milano, via Bonardi, 3 20133 Milan, Italy

**Keywords:** Insecurity, Inequality, Poverty, Middle class, Welfare state

## Abstract

Economic instability, social changes, and new social policies place economic insecurity high on the scholarly and political agenda. We contribute to these debates by proposing a new multidimensional, intertemporal measure of economic insecurity that accounts for both its multiplicity and its dynamism. First, we develop three theory-driven, multidimensional measures of economic insecurity. Principal Components Analysis validates the measure. Second, we develop a dynamic approach to insecurity, using longitudinal data and a newly revised headcount method. Third, we then use our new measures to analyze the distribution of insecurity in Europe. Our analysis shows that insecurity is widespread across Europe, even in low-inequality, encompassing welfare states. Moreover, it extends across income groups and occupational classes, reaching into the middle classes.

## Introduction

In the postwar period, economic growth, welfare states, and social dialogue supported the expansion of economic security across social classes in many European societies. But since the 1990s, the deregulation of labour markets and the emergence of new social risks have been eroding the social foundations of such stability, potentially spreading economic insecurity into to the middle classes. The recession of 2008–2013 and the ongoing COVID-19 crisis may be reinforcing these trends, further spreading economic insecurity higher up the class ranks.

Spreading economic insecurity is surprising for Europe, where the debate tended to focus on poverty trends and social exclusion (Förster & Vleminckx, 2004; Cantillon and Vanderbroucke, 2014; Mood, 2015) rather than the “middle-class squeeze” as in the United States (Frank, 2013; Hacker, 2008; Pressman, 2007; Scott & Pressman, 2011). For instance, scholarship on the 2008–13 recession stresses its impact on poverty risks and hardship (Atkinson et al., 2017; Matsaganis & Leventi, 2014), with scarce attention paid to the consequences for the middle class. Such different emphasis may reflect the assumption that the redistributive capacity of European welfare states insulates middles classes from financial precarity (Dallinger, 2013). Thus, in Europe there is still relatively little comparative research on economic insecurity specifically (Fouarge & Layte, 2005; Jenkins et al., 2012; Kenworthy, 2013; Rohde et al, 2014; Whelan, Russell, et al., 2015; Whelan et al., 2017; Cantó et al., 2020).

This paper brings problems of economic insecurity into the European debate, by providing a new multidimensional measure of economic insecurity based on longitudinal survey data. Building on the pioneering work of Gornick and Jaentti (2013), Whelan, Russell, et al. (2015) and D’Ambrosio (2018), we develop a new measure to capture the substance of economic insecurity while distinguishing it from traditional measures of permanent poverty and material deprivation. Following Osberg (1998) and Hacker et al. (2014), we focus on the objective aspects of insecurity. A principal-components analysis (PCA) supports the measure. Second, we conduct an *inter-temporal analysis* (Alkire et al., 2014) to capture the dynamic and transitory character of economic insecurity. We elaborate a revision of the AF (Alkire and Foster) headcount approach identifying “insecurity spells”. Third, we use longitudinal data from the EU Statistics of Income and Living Conditions (EU-SILC) to describe the distribution of medium-term (2–4 year) economic insecurity in the period 2007–2012. We find that in a crisis period insecurity spells varied in frequency and duration across countries in Europe, but were present across welfare regimes and varieties of capitalism. We then show that insecurity extends well up the class and income ranks.

The paper is structured in the following sections. After this introduction, Sect. 2 presents our multidimensional, dynamic approach to insecurity. Section 3 elaborates the methods used to capture economic insecurity. Section 4 presents a PCA by which indexes of insecurity are clearly distinguished from material deprivation indexes, and elaborates these results through a headcount method accounting for insecurity spells. Section 5 shows the distribution of economic insecurity across countries, classes, and income groups. Section 6 concludes the paper with a summary and broader discussion of the results, including the limitations of our analysis.

## Background

The concept of economic insecurity has been proposed to capture significant threats to the financial sustainability of households, which may be more widespread than social exclusion or income poverty (Osberg, 2018; Rohde & Tang, 2018). It identifies a condition different from material deprivation. While deprivation consists of a current shortage of essential resources for survival of families, economic insecurity rather reflects the over-time instability of economic situations. For example, an OECD report defines economic insecurity as “a significant downside economic risk—i.e. a hazard or danger—looming in the individuals’ economic future, which they are unable to adequately insure against or avoid or ignore” (Osberg, 2015: 5). This distinction is similar to that between latent and manifest vulnerability (Spini et al., 2017).

Various concepts and measures have been proposed to identify the peculiar aspects of economic insecurity. Some define economic insecurity as a high risk of losses (Western et al., 2012), adverse shocks (Bossert & D’Ambrosio, 2013) or downside hazards (Osberg, 2015). One influential line of work stresses instead the difficulty of households in covering standard expenditures (Whelan, Nolan, et al., 2015) or unexpected expenses (Brunetti et al., 2016). According to some authors (Angel & Heitzmann, 2015; Davydoff et al., 2008), insecurity means the incapacity of households to meet their financial commitments.

Others associate insecurity with protection against economic losses offered by “buffers” such as private wealth (Bossert and D’Ambosio, 2009), liquidity (Brunetti et al., 2016) or state protection (Hacker et al., 2014). This approach fails however to directly measure economic insecurity in itself.

Finally, according to D’Ambrosio and Rohde (2014), a measure of insecurity should ideally be forward looking, to capture perceptions of risk (see also Osberg, 2015; Mau et al., 2012). Rodhe and Tang (2018), for example, define insecurity as a sense of stress or anxiety that is associated with an uncertain financial future. There are however studies pointing out the weak association between objective and subjective aspects of well-being (Jahedi & Méndez, 2014; Krueger & Schkade, 2008), and a need for further investigations of the causal link between retrospective experiences and future expectations.

Moreover, while some of these studies propose unidimensional indicators of risk, others converge on the idea that economic insecurity is a multidimensional concept, requiring more complex indicators (Christelis et al., 2009; Rohde et al., 2015, 2016; Romaguera de la Cruz, 2017, 2020; Whelan, Nolan, et al., 2015; Whelan, Russell, et al., 2015; Whelan Nolan et al., 2017; Cantó, 2020). Many such indicators have been proposed, including both subjective and objective aspects, and both retrospective and forward-looking aspects (Rohde et al., 2015; Romaguera de la Cruz, 2017, 2020; Bossert et al., 2019; Cantó, 2020).

This paper builds on this debate by proposing a multidimensional conceptualization of economic insecurity. We propose a conceptual decomposition of economic insecurity, which we then evaluate empirically with longitudinal survey data. Differently from other studies, we focus on an objective, retrospective measure of economic insecurity. While this is a partial representation of insecurity, it does provide an expanded picture of the exposure of the European population to financial problems threatening household sustainability. While our approach omits prospective evaluation of insecurity, we note that fear of future adversity often results from past experience.

A second contribution of our measurement of economic insecurity is inter-temporality. Time represents an essential element of insecurity (Western et al., 2012). While studies on income shocks or poverty trajectories have shown the importance of a dynamic approach to insecurity, we still lack an approach that combine multi-dimensionality and inter-temporality (see Romaguera de la Cruz, 2017, 2020 for an exception).

Based on such assumptions, we conceptualize economic insecurity as a situation in which the financial sustainability of the household is temporary threatened. Financial sustainability refers to the household’s ability to manage financial resources in order to meet its needs throughout various life course stages and economic conditions (Hira, 2016). Threats can be due to a wide range of factors including life course events, labour market difficulties, or health strains. To capture the substance of this definition, we therefore need: (1) to decompose economic insecurity into its main dimensions, and (2) to incorporate the inter-temporal dynamics of insecurity into a comprehensive measure.

### Decomposing Economic Insecurity

Though there is no consensus on a specific objective measure of insecurity, a few dimensions of financial insecurity regularly appear in existing measures as shown above. We identify three main dimensions: (1) exposure to temporary poverty as result of income downward volatility; (2) financial strain of the households; and (3) incapacity of households to meet their financial obligations and consequent their over-indebtedness.

#### Temporary Poverty as Result of Income Downward Volatility

In the US, much attention has recently focused on large income drops or short-term income downward variability (Western et al., 2012; Hacker et al., 2014; Osberg, 2015; Nau and Soener, 2019), while in Europe increasing attention focuses on the impact of downside risks on well-being. Income volatility has thus been mainly analyzed in terms of poverty risk. Through long-term sequence analysis, the traditional approach to poverty has tended to focus on households living in permanent poverty (Vandecasteele, 2010, 2011). A “Beveridgean perspective”, whereby problematic situations are identified by reference to a collectively fixed threshold such as the poverty line, has been privileged, distinguishing these approaches from analysis of income drops. Moving to a dynamic assessment of poverty, a newer approach has tried to capture the increasing number of households facing temporary poverty situations, characterized by ups and downs around the poverty line (Whelan et al., 2003). Already the classic research of Bane and Ellwood (1986) showed that income volatility was very high in the US, and that individuals in permanent poverty were a very small minority of the total population. More recently, Sandoval et al. (2009) consider the number of spells below the poverty level, and find that in the US poverty is becoming more transitory, but its risk is becoming more widespread. A strong association between poverty risk and paid labor (Cantillon and Vandenbrouke, 2014) corroborates the idea that *temporary poverty* has increased as consequence of higher unemployment, the spread of temporary work and precarious employment in many European countries.

#### Financial Strain

Research also demonstrates the economic strain of households experiencing extremely low consumption levels, strong compression of living standards, and shortages due to illiquidity (Whelan and Maitre, 2005). In turn, Whelan et al. (2017) measure the economic strain of households facing substantial financial difficulties that are excluded from traditional indices of poverty or material deprivation. In building up measures of financial strain, Whelan et al. (*ibidem*) include not only objective over-indebtedness, but also items aimed at measuring a broader notion of unsustainable spending behavior.

#### Over-Indebtedness

The last two decades have witnessed an increase in household debt, both in Western Europe and in the US (Angel and Heitzman, 2015; Whelan et al. 2017). While household indebtedness was mainly driven by deregulation of the financial sector and proliferation of new financial instruments through 2007, since the onset of the financial crisis indebtedness has been increasingly associated with a worsening in the financial conditions of households (Fligstein & Goldstein, 2015). In recent years, scholars turned toward the role of debt in household financial insecurity (Anderloni & Vandone, 2011). Theoretical debate focuses on the neo-classical economic definition of debt as a tool for adjusting spending to expectations of future income; here, indebtedness increases economic welfare by smoothing consumption over time. However, the recent explosive growth of household debt, and especially unsecured debt (i.e. consumer credit), seems less related to consumption smoothing than to prevailing financial difficulties of overly indebted households (Jappelli et al., 2013).

### A Dynamic Approach to Economic Insecurity

Cross-sectional analysis cannot fully capture economic insecurity for many reasons. First, it cannot distinguish chronic situations from temporary shocks. Second, snapshot measures roughly distinguish poor vs. non-poor, but elide intermediate positions of transitory hardship or latent poverty (Leisering & Leibfried, 1999). Finally, households affected by contingent problems, or volatility of basic resources, cannot be identified.

We adopt a duration or inter-temporal approach (Foster, 2009) using panel data to address these shortcomings and develop a dynamic approach to economic insecurity (see also Bucks, 2011; Alkire et al. 2014). We detail the technical aspects below in Sect. 3, but here we want to clarify our general perspective and how it differs from extant research focused on dynamic poverty.

Economic insecurity focuses on downward fluctuations across multiple domains that push households into financial unsustainability. Our aim is to calculate occurrences and fluctuations of economic insecurity. First, we take a Beveridgean perspective and consider as ‘economically insecure’ the households whose economic situation temporary falls below a fixed threshold identifying financial unsustainability. We track therefore the exposure of households to insecurity spells.

Second, we hypothesize that economic insecurity is mainly related to short-term variations and is therefore separated from permanent poverty trajectories. To perform this analysis, we adopt a revised version of the headcount approach (Alkire et al., 2014) that integrates the classical AF approach (Alkire & Foster, 2011) and a spell, or duration, approach (Foster, 2009). The classic headcount approach measures multidimensional poverty by aggregating a class of different indices across multiple dimensions and over time. More specifically, it defines a unit (individual or household) as poor if it experiences scarcity of basic resources and if such scarcity is repeatedly experienced over time (number of spells). Researchers exogenously fix the thresholds indicating the multiple dimensions of poverty and time (each “dimensional cut-point” and the “time cut-point”). This approach allows the simultaneous analysis of multiple variations occurring in a limited number of spells.

## Methodological Aspects

In this section, we present our main methodological choices.

### The Headcount Method

In order to identify the dimensions to be used in the analysis, we first run a PCA on multiple items related to economic insecurity. The PCA was aimed to understand the association between items and to identify underlying dimensions. We will show that PCA results basically confirm our assumption that measures of economic insecurity are separate from those related to material deprivation, and are articulated in two independent aspects at least. We tested the robustness of our PCA results by running a confirmatory factor analysis.

After having selected two PCA factors that account for relevant dimensions of insecurity, we then calculated an additive index based on items associated with each of those factors. Building on Nolan and Whelan (2009), the value of each item has been weighted by country specific “prevalence weights”^1^ to control differences by country.

Inspired by the classic headcount approach, we proceeded as follows:

1. We fixed one dimensional cut-off for each index based on the number of items included in the index.
2. We added a further income-based indicator (not included in the PCA) to integrate short-term poverty in the analysis.
3. For each dimension, we counted the number of spells in which the households’ score is higher than the cut-off point. This summation can be expressed as:
  We thus measured change over the four-year period in each index for each household.
  $$\mathop \sum \limits_{t = 1}^{n} {\text{ dt}} = 1\quad {\text{ if i}} > {\text{k}}$$
  $$where\; d \;=\;dimensional \;insecurity; \;t\;=\;year;\;i\;=\;value\; of\; the\; dimensional \;index \ ;\ k=\ dimensional\ cut\ off\ point \ ;\ n=numbers\ of\ years\ considered.$$
4. Finally, following Bossert et al. (2012) we calculated a continuity weight to account for the aggravating condition of persistence in insecurity.

We revised the original AF headcount approach to reduce the arbitrariness that is inherent to decisions related to the cutoff points. In the AF approach, three cut-offs are usually required (Alkire et al., 2014): one to determine who is deprived in each dimension (unidimensional identification), one to determine who must be identified as poor in the multidimensional space (multidimensional identification), and the third to identify the duration of over time (duration identification). All such parameters are the result of choices made by the researcher. While Alkire and Foster (2011) claim, following Sen (Anand & Sen, 1997), that the choice of parameters (cutoffs and weights) is always a value judgement, and that their approach allows flexibility and adaptation to different institutional or operational circumstances, it has been shown, on the other hand, that normative choices of cut-offs can impact the outcomes (see for example Aaberge & Brandolini, 2014). As it has been also pointed out (Wagle, 2014), in the case of multidimensional indexes, such arbitrariness multiplies. Therefore, we argue that the accumulation of decisions related to three cut-offs would undermine the final results. Moreover, in this paper we are more interested in the decomposition of the different forms of insecurity than in providing an aggregated measure. Therefore, we only defined a cut-off for each dimension, while we did not define a second multi-dimensional cut-off to create a unified insecurity measure. For the same reason, we did not set a pre-determined duration cut-off. This allows showing how different one-dimensional transitory and chronic situations are distributed. Finally, while the original headcount approach introduces adjustments based on the intensity of poverty (Alkire & Foster, 2011), our method considers intensity as an additional aspect that may be analyzed separately.

Our final indexes share some key properties. First is decomposability: the overall share of insecure individuals in each dimension is equal to the weighted average of subgroups’ insecurity levels, where subgroups’ weights are their share in the considered population (for a mathematical demonstration, see Alkire & Foster, 2011). This allows us to estimate profiles of economic insecurity. Second is replication invariance: the same results are produced if the analysis is replicated on an identical sample (Alkire & Foster, 2011). Third is symmetry: if two people exchange outcomes within each dimension, total insecurity does not change. Fourth is weak monotonicity: insecurity does not increase if an improvement occurs in one item (Alkire & Foster, 2011). Fifth is weak transfer: if, in a dimension, a situation of hardship is transferred from one insecure individual to another more insecure individual, the overall insecurity level is not higher than original insecurity level (Foster et al, 2013).

### Data

To develop multidimensional, dynamic measures of economic insecurity we use the EU-SILC panel database, which provides a 4-year rotational panel for all the EU countries here considered. As our study focuses on temporary flows and short-term instances of different forms of insecurity, a time span of four years is adequate to study such variations, with the limitation that we cannot capture longer-term variability. We use data covering the period from 2007 to 2013 to analyze the impact of the economic recession on social classes across different welfare regimes. We pool data from four successive rotations: from 2007 to 2010, from 2008 to 2011, from 2009 to 2012, from 2010 to 2013. We retained only households which were interviewed for the whole 4-year period considered by each rotation.

This time span adequately covers the crisis period. Indeed, the real GDP per capita of the EU-28 countries started to fall in 2008, and did not recover to 2007 levels until 2014. Thus, all households considered in our panel experienced the crisis situation for at least one year.

We focus on the working-age population, as elderly economic insecurity is distinctly shaped by the national pensions systems and would require separate analysis. In what follows, only households whose main earner starts the period younger than 60 are included in the analysis.

Finally, we include 8 European countries as representative of different varieties of capitalism and welfare regimes: Denmark and Sweden as Nordic countries; the UK as the most representative country for the Anglo-Saxon regime; France^2^ as a Continental regime and Italy and Spain as components of its Mediterranean version; Hungary and Poland as representative of central-eastern countries.

### Data Weights and Attrition

EU-SILC does not provide longitudinal household weights. Therefore, we use the cross-sectional household weights provided in the EU-SILC for the first spell of each rotation to adjust for differences in the probability of a household being sampled according to demographic differences across countries. We adjust by the effective size of each country and we estimate new weights to control the variability of the panel composition over its time span.

The four-year attrition rate is 34.3% for the overall sample, with countries’ samples ranging from 22% (Poland) to 47% (Italy), with two exceptions: Denmark, where register data grants no attrition over the time period (100% of households responding for 4 years), and United Kingdom, with attrition up to 60%. However, the absolute number of households included in the British panel is still high.^3^

We addressed attrition by estimating, for each household included in the first wave, its probability of remaining in the panel for the full rotation. Variables concerning household typology, social class structure, the family income and household’s main earner age and education level were included in the regression model run at the country level. Results were used to generate Inverse Probability Treatment Weights (IPTW), which were applied to all households in the panel. IPTWs were then interacted with the original cross section household weights for spell 1 to generate a new weight.

To address over-time changes in the composition of SILC households, we used the shared weights method (Latouche & Naud, 2001), which takes individuals moving in/out of households into account to adjust the IPTWs.

### Lack of Synchronicity Among Income Data and Other Information

EU-SILC income data refer to the calendar year preceding the year of interview, in all countries analyzed here except the UK. Economic theory on the impact of income variations on household expenditures or financial problems is still inconclusive, ranging from assumptions that short-term income changes do not greatly affect expenditures given consumption smoothing, to “rule-of the thumb” theories that households can spend only what they have just earned (Jappelli & Pistaferri, 2006). Others find that short-term income changes do not substantially alter either consumption level or available liquidity (Meghir & Pistaferri, 2011). Thus, we conclude that there is little evidence that present-year income affects economic insecurity more than previous-year income, and so in all analyses we retain all four waves of data.

## Measuring Economic Insecurity: A Three-Indexes Measure

### The Specificity of Economic Insecurity: PCA Results

Measures of economic insecurity have been usually included in multiple deprivation indexes (Nolan & Whelan, 1999), which can mask specificity of this concept.

In previous research, multiple deprivation has been identified through multidimensional measures able to capture the households’ inability, due to financial constraints, to obtain a wide range of consumptions, facilities, and social conditions “generally regarded as acceptable in the community” (Nolan & Whelan, 1999, 2011). In this stream of analysis, multiple items have been combined with the aim of defining a unique measure of deprivation, on the assumption that a plurality of dimensions can be combined together in one single index. The same approach has been adopted by the European Union, which has included a multiple deprivation indicator in the official set of statistical measures supporting the Europe 2020 strategy (Eurostat, 2016; Guio et al., 2009).

Only a few attempts have been made to unpack large multidimensional deprivation indexes to identify distinct aspects and measures (Whelan Nolan et al., 2015, 2017). However, more work remains. Here, we clarify this distinction to capture the specificity of economic insecurity.

To test the hypothesis that economic insecurity is clearly distinct from material deprivation, we conducted an exploratory Principal Components Analysis^4^ by considering a large range of multiple qualitative items. Following Whelan, Nolan, et al. (2015), all available items included in the EU-SILC survey about the financial conditions of households were used in this analysis.^5^ Given 14 dummy variables, three statistically independent factors (we used a Varimax rotation) explain 69% of the total variance.

Below we describe the PCA solution, which is shown in Table 1.

**Table 1:** Principal Component Analysis (PCA): matrix of factor loadings

	Comp 1	Comp 2	Comp 3	*Unexplained*
Washing machine			0.5344	*0.275*
Car			0.4177	*0.404*
PC			0.3554	*0.417*
Colour TV			0.4749	*0.381*
Arrears rent		0.5183		*0.288*
Arrears bill		0.4327		*0.314*
Arrears loan		0.5797		*0.221*
Debt burden	0.2090	0.3442	− 0.2029	*0.370*
Unexpected expenses	0.2618			*0.237*
Make ends meet	0.3855			*0.206*
Holidays	0.3767			*0.237*
Meat	0.3860			*0.317*
Keep house warm	0.4502			*0.353*
Housing burden	0.4633			*0.361*
*Variance explained*	29.7%	19.3%	20.0%	
***Confirmatory factor—goodness of fit tests***				
Root mean square test				0.050
Comparative fit index				0.903
Tucker Lewis index				0.885
Standardized root mean square residuals				0.036

*Source* EU-SILC panel. Years 2007, 2008, 2009, 2010. Countries considered Denmark, Spain, France, Hungary, Italy, Poland, Sweden, UK. Number of observations: 77,494 individuals. Note: loadings less than .2 in absolute value are not shown

*Financial strain* is our label for the first factor, which loads most heavily on items that ask respondents about their household’s inability to (1) *afford one week holiday once a year,* (2) *afford a meal with meat, chicken, fish (or vegetarian equivalent) …. every second day,* (3) *keep home adequately warm,* (4) *face unexpected financial expenses,* (5) *make ends meet, and (6) [bear the] financial burden of the housing costs. Through their strong association, these items show situations of high economic pressure and consumption compression, which endanger the* capacity of households to cope with financial demands and satisfy their own needs, which vary according to the socio-economic contexts where they live. While income-based measures show the households’ deficit in resources flows or stocks, our index of financial strain looks at consumption/expenditure, and shows the stresses faced by many households in keeping an adequate living standard.

*Over-indebtedness* is our label for the second factor, which loads most heavily on items that ask respondents about (1) *arrears [on] loan payments,* (2) *arrears [on] utility bills,* (3) *arrears [on] rent, mortgage,* (4) *heavy financial burden of the repayment of debts from hire purchases or loans.* These items predict over-indebtedness (Angel and Heitzman, 2015). Following the recommendation of a European group of experts (Davydoff et al, 2008) our index combines together (a) at least one financial commitment (arrears) and (b) one financial commitment perceived as “heavy” (a burden). While the over-indebted household has been conceptually defined as “one whose existing and foreseeable resources are insufficient to meet its financial commitments without lowering its living standards” (Fondeville et al., 2010, p. 4), more practical measures have been adopted in empirical research. According to previous research, subjective or objective indicators considered separately show strong limitations due to different individual judgment of what “difficulty” means, and huge variability in national legal regulations governing late payment. Therefore, a mixed strategy combining subjective and objective items seems to be preferable (Whelan, Nolan, et al., 2015).

*Material deprivation* is our label for the third factor, which loads most heavily on items that ask respondents whether they (1) *can’t afford car,* (2) *can’t afford PC,* (3) *can’t afford washing machine,* (4) *can’t afford color TV.* These items measure the household resource endowment as the ability to afford durable goods. When some of these durable goods are missing due to affordability problems, we have a situation of material deprivation. These items, though some of them are under revision that can affect the headline indicator itself (Guio & Marlier, 2013; Guio et al., 2012), are still regularly included within currently used multiple deprivation indexes (Eurostat, 2016).

A Confirmatory Factor Analysis verified the robustness of these findings (see Table 1). Goodness of Fit tests confirm that the our PCA solution is adequate for the whole sample. Tests were run also at the country level with the same positive results.

The PCA results align with our conceptualization of economic insecurity (discussed in 2.1). First, the PCA identifies a specific latent variable that mainly captures the permanent absence of durable goods and fundamental resources in the household. We call this “material deprivation.” This aspect, related to permanent lack of fundamental resources rather than to our concept of economic insecurity, is found to be empirically distinct from the other two PCA factors. Secondly, the PCA identifies two factors not only distinct from material deprivation, but also independent from each other. We label these two, respectively, “financial strain” and “over-indebtedness”. These two factors align closely with two aspects of economic insecurity discussed above (see Sect. 2.1). The PCA results, in showing patterns of association that distinguish independent dimensions, give empirical support to our conceptual treatment of financial strain (economic fragility and consumption compression) as distinct from over-indebtedness (inability to keep financial commitments).

### Further Dimensions of Economic Insecurity

While the PCA results support our conceptual framework (see Sect. 2.1), the dimensions identified do not consider temporary poverty as result of income volatility. In order to capture these situations, we calculated the number of fluctuations of the household equivalent income below the yearly national poverty line (60% of national median income). *We used a dynamic measure of poverty (called “temporary poverty”) and not more common volatility measures (such as income drops, for example) following our Beveridgean approach (*see Sect. 2.2*). Unfortunately, there was no way to distinguish “planned” from “unplanned” or involuntary income drops below the poverty line, as in previous work on income volatility (*Western et al. 2012*).*

### The Multi-Dimensional Indicator of Economic Insecurity

**Fig. 1:**
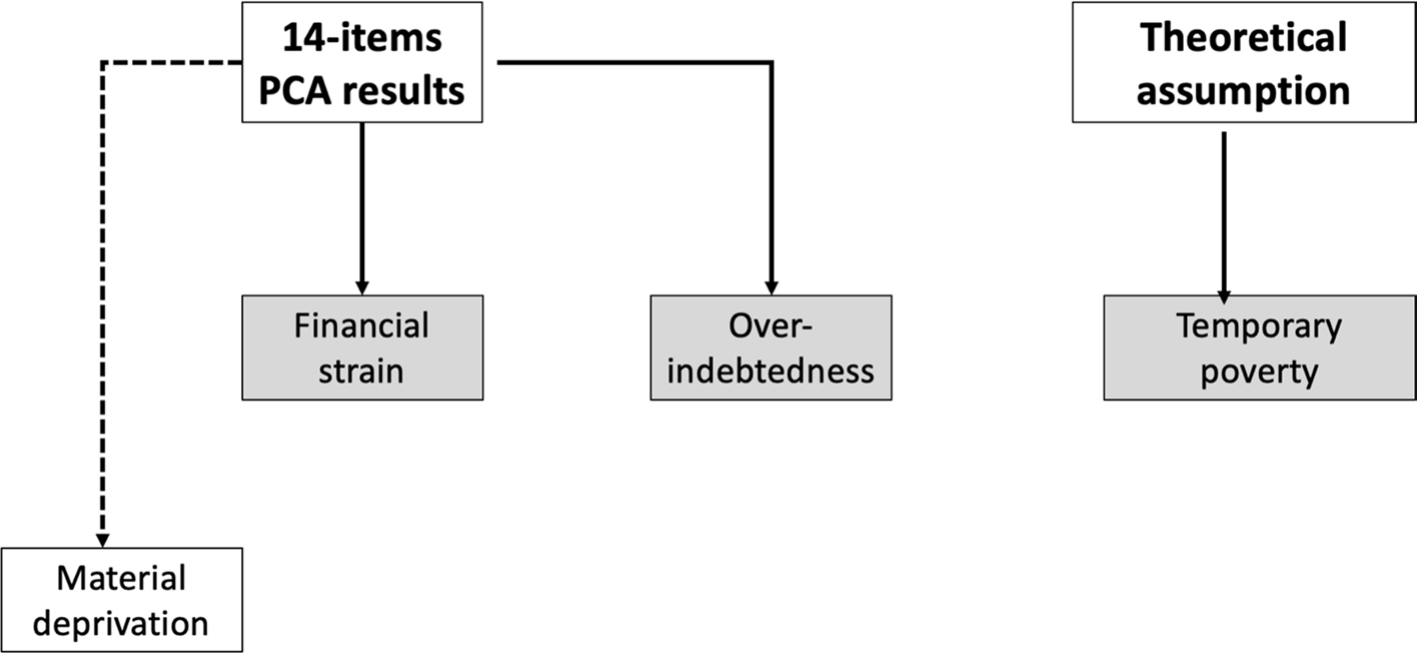
The composition of the multidimensional index of economic insecurity

*Putting these pieces together, we measure e*conomic insecurity as a combination of three distinct indexes: financial strain, over-indebtedness, and temporary poverty (see Fig. 1). Each index has been calculated by using the headcount methodology described in Sect. 3 (Alkire & Foster, 2011). For the first two indexes, following Whelan et al (2001, 2012), we assigned each item of our PCA results to a single dimension based on the criteria of the greatest association between items and the extracted factors. The assignment to only one dimension is necessary to create indexes on which the headcount method is applicable.

For financial strain and over-indebtedness, cut-off points were identified based on the number of items. Therefore, for “economic strain” (which adds 6 items) the cut-off point was established at 3, and for “over-indebtedness” (which adds 4 items) at 1.^6^ These cut-off points are applied to every year considered to identify unsecure households for each dimension. To identify “temporary poverty” the indicator for each year is represented by a dummy variable indicating if the household income is below or above poverty line.

Finally, for each dimension, spells above the cut-off points are counted over a four-year period. Table 2 reports the main statistical results of such analysis. To create an index measuring continuity, as explained in Sect. 3.3, we followed Bossert et al. (2012) and weighted our headcounts twice every time the household was below one of the cut off points consecutively rather than in separated spells. Each insecurity indexes varied therefore between 0 and 7. We see from Table 2 that “financial strain” and “temporary poverty” are the most diffuse aspects, with similar average duration and continuity. “Over-indebtedness” is obviously less diffused as this index captures only households that are explicitly unable to pay their financial commitments.

**Table 2:** Headcounts of multidimensional intertemporal indexes of economic insecurity

	Financial strain	Over indebtedness	Temporary poverty
Perc. total headcount (at least once)	31.6	17.4	28.6
Average duration (number of spells)	2.2	1.8	2.2
Percentage average duration (over max. duration)	55.0	45.0	55.0
Average continuity	3.2	2.5	3.2

*Source* EU-SILC panel: pooling of rotations from 2007/10 to 2010/13. Countries considered Denmark, Spain, France, Hungary, Italy, Poland, Sweden, UK. Number of observations: 44,683

Building on these results, we develop the next analysis in three steps. First, we analyze the time dynamic of our indexes and we look at the intersections among them. This allows us to build up *a new typology of economic insecurity*, which is composed of several categories. Second, we observe the cross-country differentiation of this typology to investigate whether and to what extent differentiated social and welfare regime contexts may affect economic insecurity. Third, we consider how households affected by different aspects of economic insecurity are distributed across diverse class groups.

### The Dynamic of Economic Insecurity

**Fig. 2:**
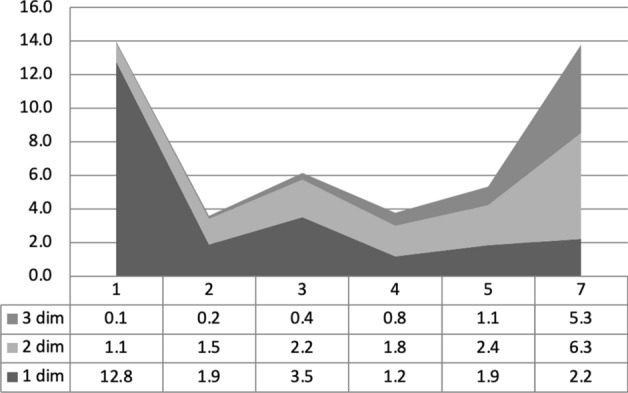
Share of households (Y-axis) in economic insecurity by weighted number of spells (X-axis) and multiple (from 1 to 3) dimensions of insecurity (total = households with at least one insecurity spell). Source: EU-SILC panel: pooling of rotations from 2007/10 to 2010/13. Countries considered Denmark, Spain, France, Hungary, Italy, Poland, Sweden, UK. Number of observations: 44,683

Figure 2 gives a sensitive representation of the relevance and characteristics of temporary insecure conditions as opposed to more chronic situations. While the Y-axis shows the share of households affected by one to three insecurity dimensions (this number only indicates the number of insecurity dimensions, whatever they are), the X-axis reports the time continuity of insecurity, ranging from 1 (only one spell) to 7 (continuous insecurity for the whole surveyed time). Households experiencing at least one spell of insecurity (whatever dimension is considered) are concentrated in two big groups: those only with one spell mostly involving one single dimension of insecurity (concentrated on the left side), and those facing many spells and mainly two or three multiple insecure conditions at the same time (shown on the right side). While chronicity very often involves a progressive accumulation of insecure conditions, many households experience only a very temporary (mostly only 1 year) critical situation affecting only one dimension.

From these results, we elaborate a typology, shown in Table 3 where different situations are dynamically described.

**Table 3:** Headcounts for different insecurity situations

	Short-term one-dimensional economic insecurity			Financial strain + over-indebtedness	Long-term multiple economic insecurity	Total
	Financial strain	Over-indebtedness	Temporary poverty			
Headcount ratio	**9.9**	**3.2**	**10.3**	**4.9**	**18.3**	**46.6**
*Continuity index*	*Column per cent over the number of households in insecure situation*					
1	53.0	70.8	50.9	9.6	3.7	30.0
2	9.8	4.7	7.6	11.1	6.3	7.7
3	15.5	13.4	15.2	17.0	9.8	13.2
4	4.9	2.2	5.9	13.7	10.6	8.1
5	7.7	6.2	8.7	15.8	14.8	11.4
7	9.1	2.8	11.7	32.8	54.9	29.6
Total	100.0	100.0	100.0	100.0	100.0	100
*Continuity index*	*Column per cent over the total number of insecurity spells*					
1	22.0	39.4	19.5	2.2	0.7	7.9
2	8.1	5.2	5.8	5.0	2.3	4.0
3	19.3	22.3	17.5	11.4	5.4	10.4
4	8.2	4.8	9.1	12.3	7.8	8.5
5	16.1	17.3	16.7	17.7	13.5	15.0
7	26.3	10.9	31.4	51.4	70.3	54.3
Total	100.0	100.0	100.0	100.0	100.0	100.0

*Source: EU-SILC panel, pooling of rotations from 2007/10 to 2010/13. Countries considered Denmark, Spain, France, Hungary, Italy, Poland, Sweden, UK. Number of observations: 44,683*

In the first group, we have many households experiencing short-term, one-dimensional economic insecurity: 9.9% experience *financial strain*, 3.2% face *over-indebtedness*. For most of these households such situations occur only temporarily. Many other households (10.3%) experience income fluctuations sufficient to temporarily bring them under the poverty threshold, but fewer signs of financial stress (see households characterized by “temporary poverty”). For such households low income apparently coincides with no significant financial or consumption pressure: a situation described as “integrated poverty” (Böhnke, 2008; Paugam, 1996). In sum, we observe that 23.4% of households have been affected by a short-term, one-dimensional form of economic insecurity.

In an intermediate position there is a smaller group (4.9%) of households that combine *financial strain and over-indebtedness*, but not poverty spells: these are households in strong financial difficulty due to costs that they are unable to meet even though income has no significant downward fluctuations in the observed time span. Furthermore, very often such financial difficulty occurs on a temporary basis, with significant long-term consequences only in a few cases.

In the second group, a large share of households (18.3%) experiences a prolonged trajectory of multiple insecurity, involving frequent fluctuations under the poverty line combined with cumulated financial strain or over-indebtedness. These multiple critical situations do not often occur synchronically (in the same year), but, more frequently, households shift from income poverty to illiquidity problems or over-indebtedness in a vicious circle made of income shortage and consequent strong consumption compression and high financial vulnerability. Chronicity and multi-dimensionality describe these households, which are defined as falling in a “*long-term multiple economic insecurity*”.

Table 3 shows also the over-time distribution of the various categories. As expected, one-dimensional *financial strain, over-indebtedness* or *temporary poverty* are mainly conditions affecting households for only one year or two separate years. On the contrary, households experiencing multiple forms of economic insecurity are more likely to be affected for three or four spells.

If the headcount is calculated by years (weighted by continuity) in insecurity rather than by number of households (see Table 3, second half), the weight of chronically insecure households becomes higher, consistent with previous results by Bane and Ellwood (1986). Table 3 shows that trajectories characterized by *long-term multiple insecurity* count for 54.3% of total years even though they involve only 29.6% of households. On the other hand, 30% of households experiencing transitory (one spell) economic insecurity accounts for just 7.9% of total years. We have therefore a high concentration of difficult spells in a relatively limited number of households on the one hand, and a low diffusion of difficult spells in a large number of households on the other. This second group is not affected by high risk of poverty (Mood, 2015), but experiences a form of economic insecurity characterized by difficult situations for a very limited amount of time, over the four-year period examined here.

To sum up, the number of households dealing with economic insecurity captured through this approach is very high. Over 46% of households in our eight countries underwent a period of economic insecurity within a four-year time span (see Table 3). Many suffered only temporary insecurity, which mainly affected only one dimension of their living conditions. These insecure households do not constitute a large percentage of the socially insecure in any one year, given that they are not permanently insecure. But these insecure households do constitute a large share of the population, representing around 23.4% of the total households in our eight countries.

## Cross-Country and Cross-Class Analysis

### Cross-Country Comparison

The distribution of our categories of economic insecurity across countries shows that households were differently affected by these problems across Europe during the economic crisis (see Table 4 and Fig. 2). Households in Central and Eastern European (CEE) countries were more likely to be in difficult situations than households in the western part of Europe, confirming results from Cantó et al. (2020). In Hungary and Poland critical situations were remarkably concentrated in *long-term multiple economic insecurity*: a clear sign that low income, constrained consumption and financial strain are often interweaved problems in these countries, and that these situations accumulate in a great number of households. On the other hand, the share of households affected by *temporary poverty* in CEE countries was remarkably lower than in the Western European countries included here.

**Table 4:** Headcounts of insecurity types, by country

	Well-being	Short-term one-dimensional economic insecurity			Financial strain + over-indebtedness	Long-term multiple economic insecurity	Number of observations
		Financial strain	Over-indebtedness	Temporary poverty			
Denmark	77.2	2.2	4.5	10.1	1.4	4.7	2,309
Sweden	72.1	1.7	4.7	13.6	1.3	6.6	2,776
United Kindgom	56.0	6.0	2.7	13.2	4.0	18.1	3,040
France	59.5	7.5	4.1	10.3	4.5	14.1	8,689
Spain	46.7	10.3	3.2	11.6	5.7	22.5	6,906
Italy	47.3	13.7	2.3	7.9	5.9	22.9	8,442
Poland	47.2	16.7	3.0	8.3	4.4	20.5	7,535
Hungary	37.8	23.9	3.1	3.2	12.0	20.1	4,986
Total headcount of countries	53.3	9.9	3.2	10.3	4.9	18.3	44,683

*Source: EU-SILC panel, pooling of rotations from 2007/10 to 2010/13*

In Western European countries, the share of *long-term multiple economic insecurity* was very low, and lowest in Sweden and Denmark. Mediterranean countries showed higher levels of *long-term multiple economic insecurity*, reflecting the strong impact of the economic crisis on these countries (Petmesidou and Guillén, 2017). Finally, one-dimensional *temporary poverty* predominated in the UK and Spain, but it was comparatively very high both in Sweden and Denmark. This fact may confirm the idea that risk of income volatility leading to poverty (lasting for not more than 1 year) is higher in countries with lower inequality and higher welfare protection. In 2012, indeed, the Gini index in Denmark (0.25) and Sweden (0.27) was the lowest among our countries (OECD Income Distribution Database). Moreover, in these countries, minimum income programs enabled the temporarily poor to quickly recover (Kangas & Kvist, 2013). Finally, *financial strain* was higher in Mediterranean countries, France and the UK.

**Fig. 3:**
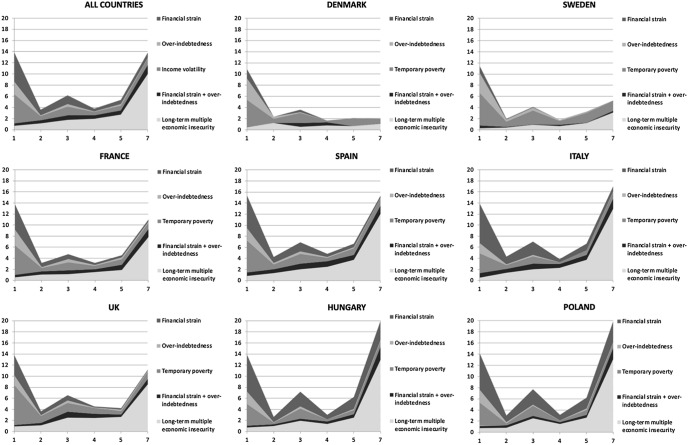
Area plots of insecurity headcounts, by country. Source: EU-SILC panel: pooling of rotations from 2007/10 to 2010/13. Countries considered Denmark, Spain, France, Hungary, Italy, Poland, Sweden, UK. Number of observations: 44,683

In sum, short-term economic insecurity was widespread in crisis Europe, although its prevalence varied systematically across countries. These cross-national differences, detailed in Table 4, are summarized as area plots in Fig. 3. The higher concentration of transitory economic insecurity in Continental countries may reflect a marked dualization in the labor market, the spread of unstable employment positions in this area as consequence of the economic recession, and higher exposure of households to risks related to financialization. On the other hand, the geographical distribution of long-term multiple insecurity was much more differentiated, with higher concentration in CEE and Mediterranean countries. In the figure, we observe a clear U shaped distribution in CEE and Mediterranean countries, to show that both long-term and short-term insecurity predominate. Nordic countries were characterized by a L-shaped curve, where only transitory problems predominate. France and the UK were in a mixed position.

### Economic Insecurity and the Middle Class

**Fig. 4:**
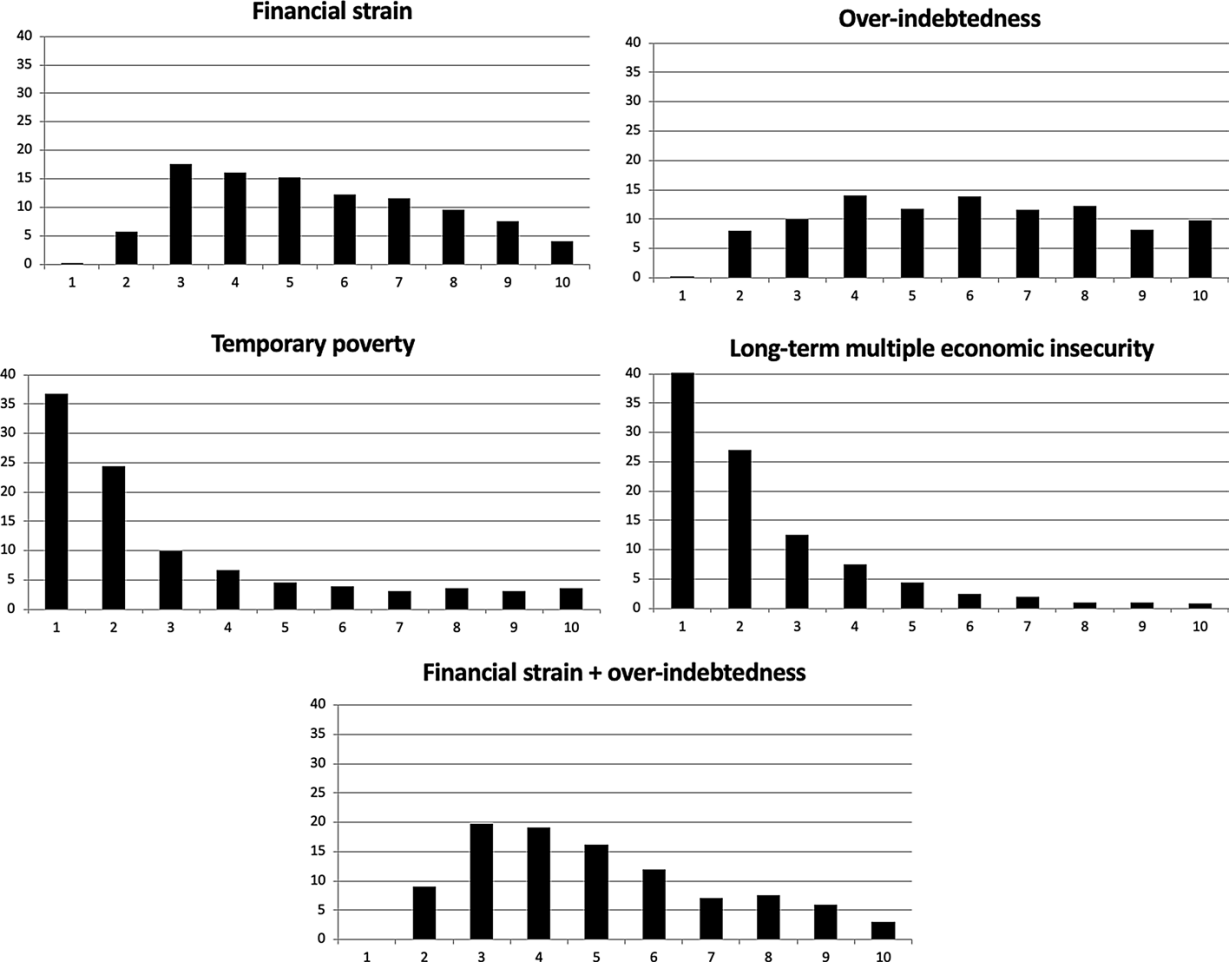
Distribution of social insecurity by income deciles. Source: EU-SILC panel: pooling of rotations from 2007/10 to 2010/13. Countries considered Denmark, Spain, France, Hungary, Italy, Poland, Sweden, UK. Number of observations: 44,683

Figure 4 shows the distribution of economic insecurity across income deciles.^7^ While long-term *multiple economic insecurity* and *temporary poverty* were unsurprisingly concentrated in the lowest three deciles, our indexes of transitory *financial strain* and *over-indebtedness* were more broadly distributed across income deciles. Thus, in the crisis, economic insecurity mainly related to consumption difficulties and indebtedness in middle-income groups, while the poorest households experienced transitory or permanent poverty.^8^ While poverty was concentrated in the poorest deciles, the lower-middle income groups (between the third and fifth decile of the income distribution) were more likely to face difficulties in their financial sustainability than low–income groups. For these groups, it was not the scarcity of income in itself, but rather expense or debt levels, that caused insecurity.

The analysis above has limited utility for sociological class analysis, since it is based on income rather than social classes. To address this limitation, we also used a simplified version of the European Socio-economic Classification (ESeC)^9^ to analyze the distribution of different types of insecurity.

**Fig. 5:**
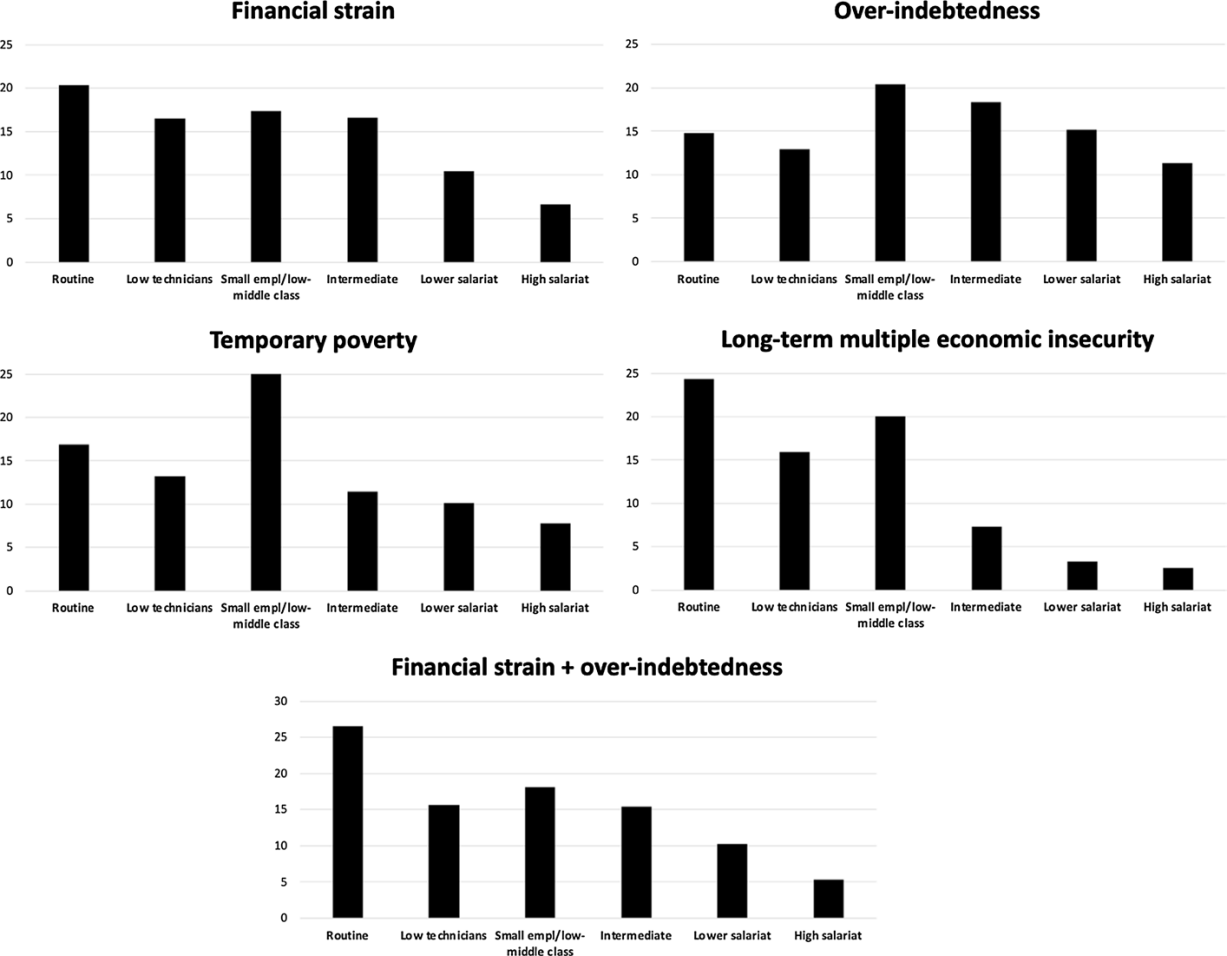
Distribution of different trajectories of insecurity by social class (ESEC classification). Source: EU-SILC panel: pooling of rotations from 2007/10 to 2010/13. Countries considered Denmark, Spain, France, Hungary, Italy, Poland, Sweden, UK. Number of observations: 44,109

Figure 5 shows that households affected by *long term multiple economic insecurity* were more concentrated not only in the working class (categories 1-2) but also in the lower middle class (category 3). Such risk was hardly experienced by households in the middle-upper and upper social classes. Other types of economic insecurity, however, did not line up so neatly with social class. For example, *temporary poverty* was significant among the lower middle class in 2007–2013, owing to the economic recession.^10^ The *financial strain* type cut most strongly across classes, and only drops significantly for the highest class. The intermediate class, which combines technicians and public employees with medium to high qualification, experienced substantial *financial strain* and *over-indebtedness*.

Looking in detail at middle class groups (categories 3-4-5), temporary *financial strain* and *over-indebtedness* were the highest and most encompassing aspects of economic insecurity. The high prevalence in all the middle-class groups gives an idea of to what extent, during the crisis, consumption compression and inability to pay debts endangered the financial sustainability of the middle class, including high-skilled professionals and managers of the “lower salariat” category. For middle-upper class people with higher qualifications, sustainability problems mean a large discrepancy between high consumption associated to their life-style and their social reputation on the one hand, and the relative scarcity or instability of material resources that were necessary to sustain that social status in crisis. For them, economic insecurity basically meant a risk of “relative deprivation” (Frank, 2013), a significant lack of resources to sustain a life-style in accordance with their social identity.

In the lower-middle class group, instead, critical financial sustainability did bring temporary poverty. This is the situation characteristic of the “petty bourgeoisie” mostly represented in the “small employer/low middle class” class. This group (composed by shop-owners, small entrepreneurs, self-employed technicians, craft workers, etc.) is traditionally characterized by relatively low education and consumption levels, and relatively high income. The self-employed, mostly working in hard-hit small-size enterprises, experienced high poverty risks, consumption compression, and over-indebtedness.

## Discussion

This paper sheds new light on the spread of economic insecurity in crisis Europe by incorporating multiple dimensions of disadvantage simultaneously, and by incorporating a dynamic perspective that reveals significant cross-country differences. Building on the work of Gornick and Jantti (2013), Western et al. (2012), and Whelan, Nolan, et al. (2015), among others, we develop an innovative approach to economic insecurity that incorporates multiple dimensions of short-term vulnerability *and* an inter-temporal perspective that allows us to identify insecurity spells. Economic insecurity is considered here as a specific combination of multiple difficulties that are dynamically and reciprocally intertwined. Moreover, our measure integrates three dimensions of economic insecurity that are often treated separately: financial strain, over-indebtedness, and temporary poverty.

The intertemporal analysis is based on a revision of the traditional headcount approach of Alkire et al. (2014). We revised this approach by using only one cut-off to identify insecurity spells instead of the original three cut-offs recommended by Alkire et al. (2014). This allows us not only to reduce the number of arbitrary decisions, but also to decompose the different dimensions and dynamics of economic insecurity. One of the main results is that we isolated short term insecurity from chronic, and potentially more dangerous, financial difficulty.

To demonstrate the utility of this approach, we analyse four waves of data from the EU-SILC, from the crisis years 2007–2013. A principal components analysis supports our argument for distinct types of insecurity, separable from material deprivation. Using this categorization, we calculate dynamic headcounts of financially insecure households, and show how these counts vary across European countries. Finally, we show that economic insecurity is broadly distributed across European households, reaching surprisingly high up the income and class ranks.

We underscore the distinctiveness of economic insecurity. First, it crisscrosses a wide range of social classes. It affects not only lower-class households but also white-collar intermediate workers and households whose income is in middle deciles. Future work should use the most recent EU-SILC data to determine whether this widespread vulnerability is structural, or confined to the recent economic crisis. Either way, this widespread vulnerability demonstrates the limited capacity of contemporary European welfare states to secure households from market volatility (Huber & Stephens, 2014).

The evidence that economic insecurity is widely distributed across the class hierarchy in Europe suggests that many households experience insecurity on a day-to-day basis that shapes their standard of living. We emphasize that this insecurity concerns not only the absolute amount of available resources, but also the stability of resource flows and the relation of income to consumption. Economic insecurity does not just increase the risk of poverty or material deprivation. Rather, it is a diffuse condition many households experience. Thus, economic insecurity not only causes problems for individual households, but may constitute a broader social problem in and of itself.

Many aspects of this widespread insecurity still need to be investigated. Although this paper describes the distribution of economic insecurity across European nations and households in crisis time, we have left the explanation of these patters to future work. Notably, we lack data on psychological orientations toward the future. Additionally, we need new research on the relationship between trigger events and specific insecurity situations. And, structural conditions potentially responsible for the distribution of economic insecurity need to be properly assessed. Finally, we need to analyse the consequences of insecurity, in terms of social behaviour, investments in the future, and political orientation. Short-term insecurity may have different impacts on people depending on the role played by public and private buffers. In this sense, we call for new research to understand what role may be played by broad welfare regimes and specific social policies in protecting people from insecurity and its potential consequences.

## Data Availability

Syntax used to elaborate data is available upon request.

## Footnotes

1 Prevalence weights were calculated to control by the variability of the distribution of items within each country, so that less prevalent hardships in each country contribute more to the total counting. The formula used is: [1 − (share of households reporting lacking item i/total share of households reporting lacking items included in the index)]. The value obtained is then multiplied by the number of items part of the index (n) and divided by (n−1).

2 The French SILC panel follows part of the households for 8 years. We randomly sampled only one rotation for such households.

3 Number of households remaining after attrition: Denmark 2355, Spain 6950, France 8972, Hungary 5122, Italy 8446, Poland 7588, Sweden 3303, United Kingdom 3077.

4 The PCA was run on spell 1 by considering the full sample. We used a tetrachoric correlation, which is a proper technique for estimating correlation between dichotomous variables. Varimax rotation was applied. Factors showing eigenvalue > 1 were retained, producing a 3-factor solution.

5 We included all the available items but those related to the housing conditions of households (not frequently considered in such analysis) and one specific item (telephone not affordable) that is not available for some of the analyzed countries.

6 For the over-indebtedness index, we chose 1 as a cut-off as prevalence weights applied lowered the value of two items to considerably less than 1 in many countries.

7 Income deciles are calculated for the first wave of our panel. The graph therefore shows how different types of insecurity developed over time from that starting position.

8 Country-specific analysis (not shown in the paper but available on request) confirmed the same results for all the national contexts here considered with only minor exceptions.

9 ESEC classification has been developed following Rose and Harrison (2007)’s simplified procedure. The standard 9 classes ESEC were synthetized in six classes grouping together “Small employer and self-employed occupations”, “Self-employed occupations”, “Lower supervisory and lower technician occupations” and “Lower services, sales and clerical occupations” into a “Small employers, lower middle class” group. We classify individuals considering ISCO information from the first year’s observation.

10 High poverty among small employers/lower technical class may also reflect a difficulty of the EU-SILC in measuring incomes of small employers, who show high levels of underreporting in a number of countries.
